# Supplementary figures and images for: Spatio-temporal analysis of the distribution and co-circulation of dengue, chikungunya, and Zika in Medellín, Colombia, from 2013 to 2021
Source: PLoS Negl Trop Dis. 2025 Sep 5;19(9):e0013470. doi: 10.1371/journal.pntd.0013470 (PMC12425392; doi:10.1371/journal.pntd.0013470)

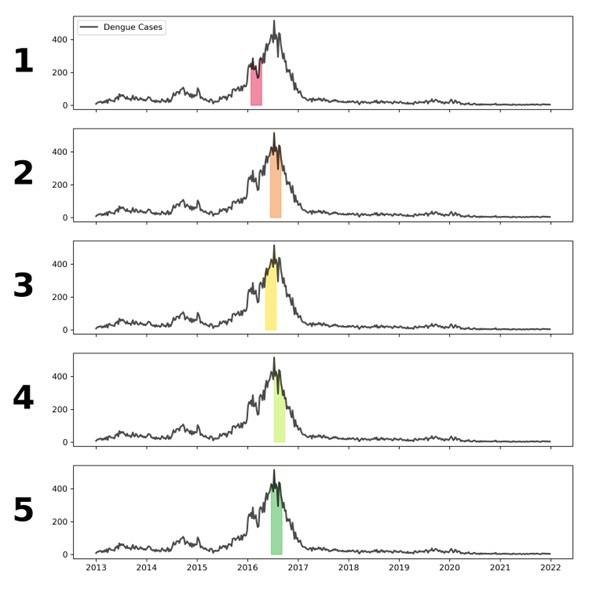

Supplement: S1 Fig — (TIF) [file pntd.0013470.s001.tif]

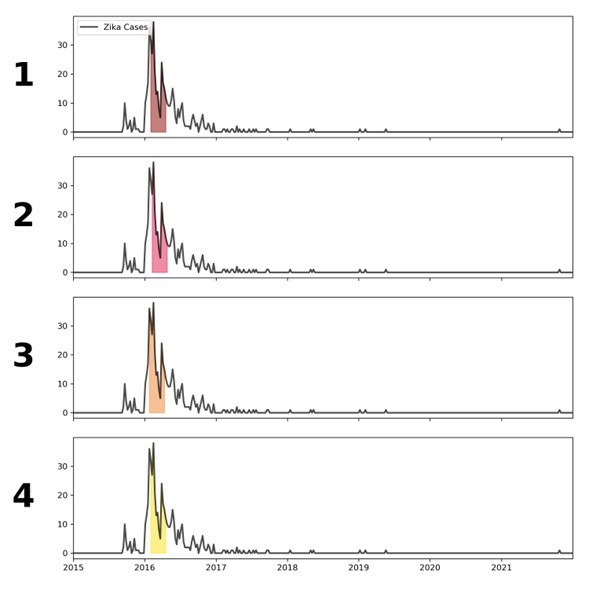

Supplement: S2 Fig — (TIF) [file pntd.0013470.s002.tif]

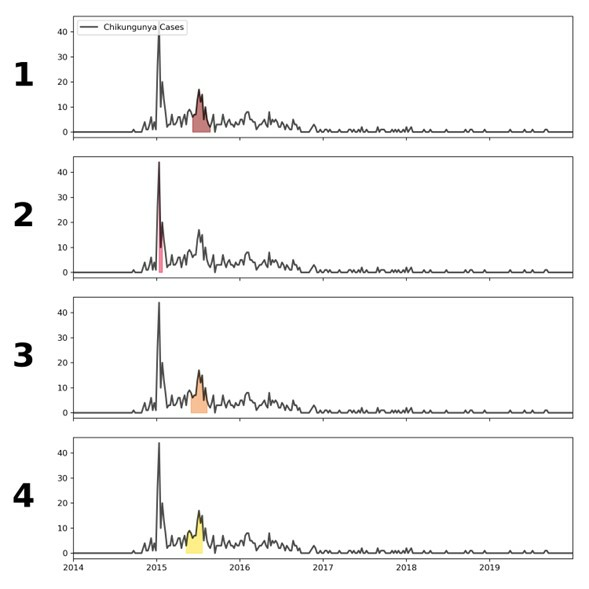

Supplement: S3 Fig — (TIF) [file pntd.0013470.s003.tif]

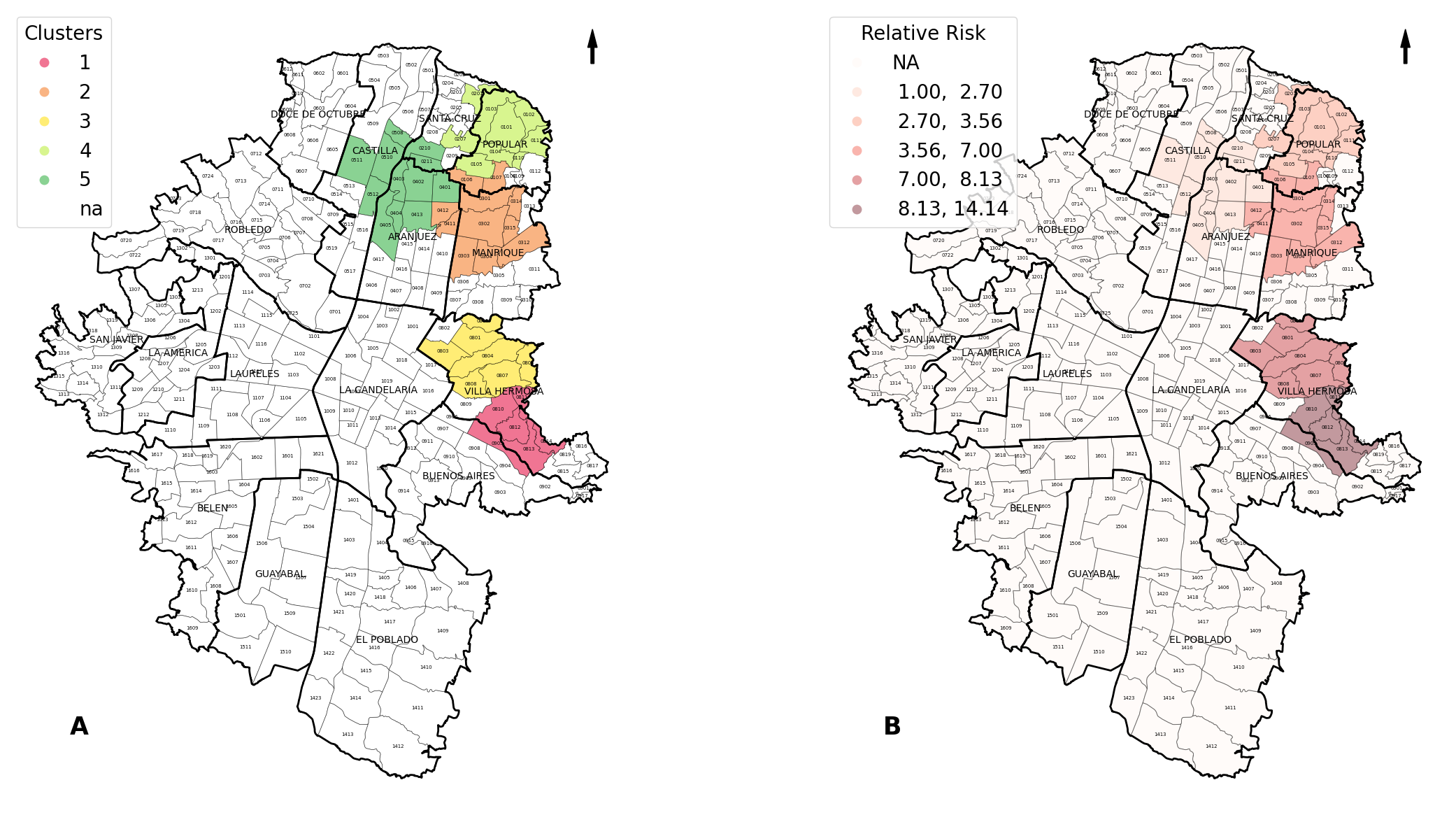

Supplement: S4 Fig — Note: The map was generated using municipality boundary basemaps from GADM (www.gadm.org) and open data from the Medellín city portal (https://www.medellin.gov.co/geomedellin/datosAbiertos/1043). (TIF) [file pntd.0013470.s004.tif]

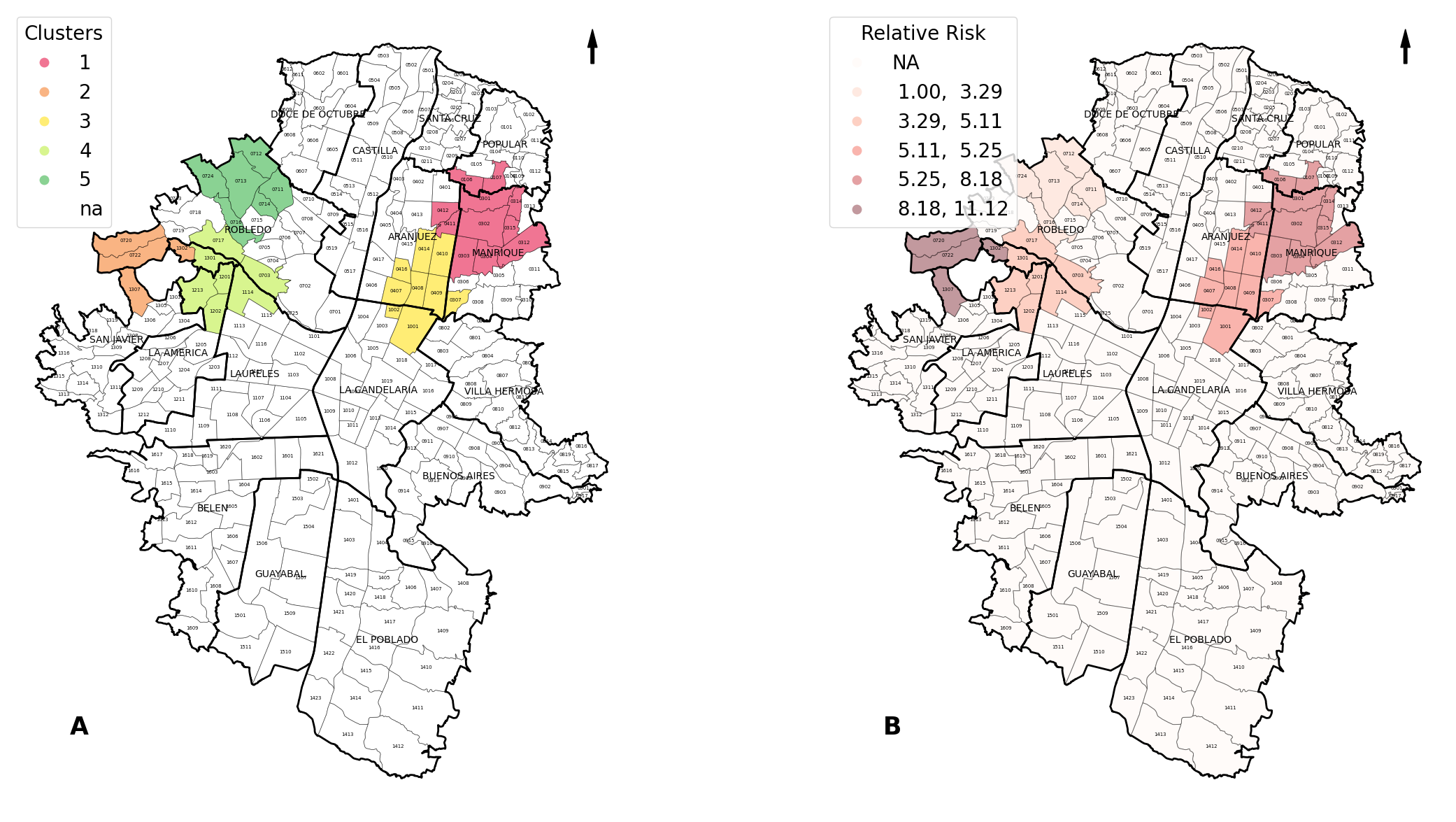

Supplement: S5 Fig — Note: The map was generated using municipality boundary basemaps from GADM (www.gadm.org) and open data from the Medellín city portal (https://www.medellin.gov.co/geomedellin/datosAbiertos/1043). (TIF) [file pntd.0013470.s005.tif]

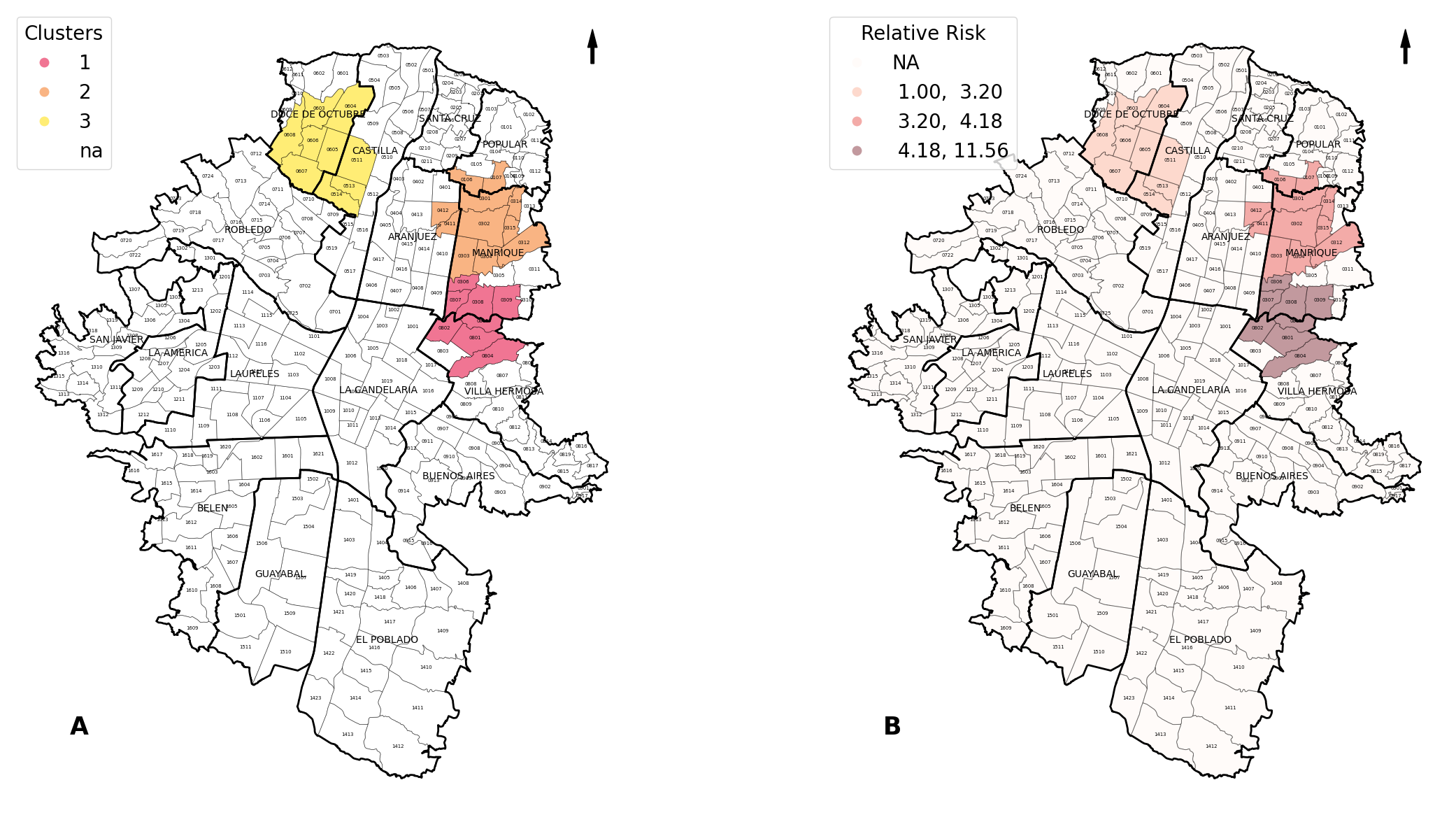

Supplement: S6 Fig — Note: The map was generated using municipality boundary basemaps from GADM (www.gadm.org) and open data from the Medellín city portal (https://www.medellin.gov.co/geomedellin/datosAbiertos/1043). (TIF) [file pntd.0013470.s006.tif]
