# Supplementary material for: Spatio-temporal analysis of the distribution and co-circulation of dengue, chikungunya, and Zika in Medellín, Colombia, from 2013 to 2021
Source: PLoS Negl Trop Dis. 2025 Sep 5;19(9):e0013470. doi: 10.1371/journal.pntd.0013470 (PMC12425392; doi:10.1371/journal.pntd.0013470)
Supplement: Attachment — Submitted filename: Response to Reviewers.docx [file pntd.0013470.s007.docx]

May 05, 2025

**Shaden Kamhawi**

Editor-in-Chief

PLOS Neglected Tropical Diseases

Dear Editor and reviewers,

Dear editor and reviewers,

We sincerely appreciate the time and effort that you and the reviewers have dedicated to evaluating our manuscript, *" Spatio-Temporal Analysis of the Distribution and Co-Circulation of Dengue, chikungunya, and Zika in Medellín, Colombia, from 2013 to 2021”.* We are grateful for the constructive feedback, which has helped us refine and strengthen our study.

Below, we present a detailed point-by-point response to each of the reviewers' comments. For clarity, all revisions in the manuscript are highlighted in turquoise blue.

We hope that our revisions and responses meet the reviewers’ expectations and that the revised manuscript is now suitable for publication in *PLOS Neglected Tropical Diseases*. Please do not hesitate to contact us if any further modifications are required.

**Reviewer 1**

| **Comment** | **Response to Reviewer** |
| --- | --- |
| **Methods** |  |
| The study does not present a clear testable hypothesis. It is clearly articulated with described goal, though. | The hypothesis was refined to align with the stated research objective. |
| According to description, is it correct to assume that only clinical cases were considered for the study? Please clarify | Thank you for the comment. We clarify that the study included both probable cases -defined by clinical and epidemiological criteria- or confirmed cases, which were verified through laboratory methods such as RT-PCR, ELISA NS1, or viral isolation, following the national surveillance definitions. |
| It is not well explained what is the nature of data being mapped (and provided by SIVIGILA): Is it the house of the cases? | These data correspond to the case's place of residence, and this clarification has been included in the manuscript. |
| In lines 188 -189, what was the purpose to transform cases into standard normal distribution? Visualization? What test was applied to check such distribution? In addition, if data were z-transformed, I suppose it means that data follows a normal distribution, am I right? This is particularly important to clarify as the calculations for RR are assuming that data follows a normal distribution. | This transformation was applied for visualization purposes. It does not imply that the original data follow a normal distribution, and no parametric assumptions were made in the relative risk (RR) calculations, which are based on count data and not on z-scores. |
| Line 194: What kind of discrete Poisson model was fitted? Generalized linear, Bayesian hierarchical? Mixed? | The study used Kulldorff’s scan statistics with a discrete Poisson model, implemented through SaTScan software. This model is Bayesian hierarchical. It is a likelihood-based cluster detection method |
| Explain further what "I()" means in the equation number 4. It is stated that it’s an indicator function, but it is an indicator of what? and why it is the same (1) for all windows? Multiply by 1 would mean that it makes no difference, so I don’t see the purpose of such value in the equation. | We noticed that the equation was incomplete and have now included the missing information. |
| **Results** |  |
| Figures S4, S5, and S6 which are supposedly showing spatio-temporal cluster are only showing temporal clusters (no spatial reference at all) and all of them corresponds to the same outbreak. So, they are no informative. | Indeed, these are temporal figures. We believe it is important to retain them, as they clearly illustrate the main incidence peaks for each disease, which are critical for understanding their temporal dynamics and guiding public health interventions.  Figure captions have been revised to improve clarity and avoid ambiguity.  Figures were renamed because some figures were removed. |
| **Conclusions** |  |
| Please take a look at "Summary and General Comments". | The comments were reviewed. |
| **Editorial and Data Presentation Modifications?** |  |
| The available captions for supporting figures should be more descriptive. | Thank you for your suggestion. We agree that the original captions for the supporting figures lacked sufficient detail. We have revised all figure captions in the appendices to be more descriptive and informative, explicitly indicating the content, time frame, and relevance of each figure to the analysis. |
| It is hard to elucidate the difference between neighborhood, district, and comunas. Please define them properly to ease the understanding of the spatial description of the clusters. | Thank you for pointing this out. We acknowledge that the distinction between "neighborhood," "district," and "comuna" may not be immediately clear to international readers. We have revised the manuscript to include this clarification in the "Area and Study Population" section to improve the reader's understanding of the spatial context. |
| **Major comments:** |  |
| The manuscript describes a study analyzing historical cases data and their spatial distribution. While these types of studies are locally relevant, I believe they may have limited appeal to a broader audience as it is limited only to the descriptive level. Though it is stated in the Introduction that the aim is “to identify high-risk clusters for each disease individually and collectively to understand these arboviruses' co-circulation potential”, the manuscript doesn’t sufficiently describe the importance of achieving such goal. For instance, what is the significance of describing such patterns and how can these findings inform strategies for future application? In this sense, it seems to me that further analyses should be done to not limit the study to a narrow goal. In this way, the work can in addition be more innovative and original.  There is a longstanding issue partially resolved in recent years about this type of studies dealing with the place of infection’s acquisition. Though it is not properly explained in methods, I am assuming that authors are mapping the cases’ home addresses. If so, this study is assuming that infections are taking place at cases’ households. Last evidence has shown differential distribution of vectors between households and environments other than households as well as increased risk of infection in the latter. According to this, it is important to notice that clusters are not depicting infection risk or similar, but the spatial distribution of cases. A differential distribution of human population density within the city might potentially explain such distribution. In concordance, authors didn’t describe any density-based correction. What is the probability of the cluster of cases to be explained by a locally higher human population density? In my view, addressing these issues is crucial to align the study with current epidemiological and spatial knowledge.  Finally, identified clusters correspond to the same temporal outbreaks. So, in my understanding those are showing a one-time higher density of cases. I would expect for some cluster to be repeated in time and space, so preventive actions can be applied later. Since there are no spatial patterns repeated over time, it is hard to extract information that can be translated into something actionable. I wonder if a sensitive analysis was done to achieve highest performance of the spatial modeling. | Thank you for your thoughtful and detailed feedback. We have revised the manuscript to better highlight the relevance and implications of our findings. While this is a descriptive ecological study, we clarified in the Introduction and Discussion how identifying spatio-temporal and multivariate clusters supports targeted surveillance and intervention strategies in urban settings like Medellín, where arboviral co-circulation and socioeconomic disparities intersect.  We acknowledged in the Methods and Limitations that cases were geocoded by residence, which may not reflect the exact place of infection. However, residential clustering remains useful for planning community-based actions. Regarding population density, we clarified that the SaTScan Poisson model adjusts for population at risk, but we now explicitly note the need for finer intra-urban density corrections.  Finally, we addressed your comment on temporal recurrence and model robustness by clarifying that cluster detection parameters were tested for consistency, and that future studies should incorporate formal sensitivity analyses and explore recurrent patterns over longer periods. |

**Reviewer 2**

| **Comment** | **Response to Reviewer** |
| --- | --- |
| **Methods** |  |
| -Is the study design appropriate to address the stated objectives? Partially. The methods are not clearly described, so the results are not reproducible. | Thank you for your observation. We have revised the *Methods* section to improve clarity and reproducibility. We now provide more specific details on:   - Case definitions and inclusion criteria - Geocoding procedures and software used - SaTScan configuration: window sizes, temporal and spatial parameters, number of simulations, and statistical assumptions - Calculation of relative risk and confidence intervals   These clarifications aim to ensure that our methods can be reproduced by other researchers. |
| Is the population clearly described and appropriate for the hypothesis being tested? No, it is not made clear from the outset that the data used correspond to potential cases and not proven cases, and the implications that this may entail. | Thank you for your observation. We have revised the study population and methods sections to explicitly state that the analysis is based on reported cases, including both probable and confirmed cases as defined by national surveillance protocols. We also added clarification on the implications of including suspected cases in the limitations section. |
| Were correct statistical analysis used to support conclusions? It is not easy to know since they do not adequately describe the methods used. | We have revised the Methods section to provide a clearer and more detailed description of the statistical analyses used to support our conclusions. |
| **Results** |  |
| Does the analysis presented match the analysis plan?  No completely. There are discussed results that cannot be inferred from the analyses. For example, the occurrence of waves of cases in dengue, Zika and chicungunya, the lack of seasonal patterns in Zika and chicungunya, and the seasonal patterns in dengue. | Thank you for your important comment. We agree that some temporal interpretations, such as epidemic waves and seasonality, were based on visual inspection of the time series rather than formal trend or seasonal analysis. We have revised the results and discussion sections to clarify this point and removed any overstated conclusions that are not supported by the methods applied. |
| Are the results clearly and completely presented? No, it is not easy to follow the results, the figures are lightly mentioned in the text. There are many supplementary figures that are not properly described. | The number of supplementary figures was adjusted. Figures 4, 5, 6, 7, 8, and 9 were retained, while six others were removed. The retained figures have been expanded with more detailed descriptions. |
| Are the figures (Tables, Images) of sufficient quality for clarity? No, there are repetitive figures, the quality is very poor, so it is not possible to read what is written. The labels are not appropriate and the northern array is incorrect. | The figures were reorganized according to the reviewers’ recommendations. Labels and north arrows were corrected and standardized. |
| **Conclusion** |  |
| Are the conclusions supported by the data presented? No, the conclusions are too general and most of them were already known. There are no adequate comparisons with other studies already conducted. The author merely mentions other studies without putting them in context and discussing the implications related to his work. | Thank you for this important comment. We have revised the Conclusions section to ensure that they are directly supported by our findings. We also included a more meaningful comparison with previous studies and discussed how our results contribute to the understanding of intra-urban arbovirus transmission in endemic urban contexts such as Medellín. |
| Are the limitations of analysis clearly described? Partially. The limitations of the data are presented in the discussion section but not the limitations of the methods. | Limitations section to include methodological limitations, such as assumptions of the scan statistic model, the ecological nature of the analysis, and the absence of formal temporal or seasonal modeling. These additions clarify the scope and constraints of the study’s analytical approach. |
| Do the authors discuss how these data can be helpful to advance our understanding of the topic under study? In a very general way, since they do not analyze the meaning of their results and what specific factors give rise to these clusters. | Thank you for this valuable comment. We have revised the Discussion section to better analyze the meaning of the identified clusters and to propose plausible explanatory factors -including socioeconomic vulnerability, urban density, vector adaptation, and timing of virus introduction- that may underline their spatial and temporal distribution |
| Is public health relevance addressed? Again, in a very general way. It is necessary to further understand the results, probably by carrying out other analyses that relate these clusters to socioeconomi, environmental, etc. factors. | Thank you for your comment. We have expanded the Discussion to strengthen the interpretation of our findings from a public health perspective. While our current design is descriptive, we now more clearly articulate how the identified clusters reflect underlying social and environmental inequalities, and we suggest future studies to explore these associations through multivariable and inferential approaches. |
| **Summary and General Comments** |  |
| Salazar Flórez et al.'s study on dengue, Zika and chicungunya in Medellín, Colombia, identified spatial patterns, with five dengue, four Zika, and four of Chikungunya clusters concentrated in the northeast of the city, as well as high-risk clusters with for all three diseases. While the topic is crucial for priorizing intervention areas, the analysis primarily reiterates previously studies without providing novel insights. The study falls short in several aspects: temporal dynamics lack robust time series analysis for statistically significant seasons or trends; cluster detection methodology is insufficiently described, hindering reproducibility; spatial results are not thoroughly examined; and environmental and socioeconomic factors are overgeneralized without considering local conditions and interactions. These limitations suggest that the manuscript, in its current form, is unsuitable for publication. To enhance its relevance to academia and society, I suggest: i) conducting a more rigorous analysis that incorporates variables explaining the observed patterns, ii) considering local conditions and factor interactions in the analysis of environmental and socioeconomic influences, iii) Providing a more detailed description of the cluster detection methodology to ensure reproducibility, iv) performing a comprehensive time series analysis to support temporal dynamics conclusions, v) analyze the results in the context of previous research in the topic. | Thank you for your detailed review. We appreciate your observations and have made substantial improvements to the manuscript to address each of your concerns:  Temporal analysis and seasonality: We clarified that the observed epidemic peaks were identified through visual inspection, not formal time series modeling. We revised the text to avoid overinterpretation and acknowledged this as a limitation, suggesting future studies apply statistical trend and seasonality analyses.  Methods: The Methods section was expanded to include detailed parameters used in SaTScan (model type, spatial and temporal window size, population limits, minimum cases, and number of simulations), ensuring reproducibility.  Spatial results:  We improved the Discussion by analyzing possible cluster formation, including population density, socioeconomic vulnerability, entomological risk, and heterogeneous Wolbachia implementation.  Environmental and socioeconomic factors: We replaced general statements with specific indicators (e.g., Life Condition Index, stratification levels) and incorporated local references to strengthen contextual interpretation. |
| **Summary and General Comments** |  |
| ***Major comments*** |  |
| 2. Introduction - L116. Please provide an explanaition of why Medellín is a relevant city to conduct this analysis. | Medellín is a relevant setting for this analysis due to its status as Colombia’s second-largest city, its hyperendemic transmission of dengue, and its early experience with chikungunya and Zika outbreaks. The city’s socioeconomic heterogeneity and high population density provide a valuable context to study intra-urban arboviral distribution and guide targeted interventions. It was clarified in the text. |
| **3. Methods** |  |
| - L120-125. Please describe here that these cases include potential and proven cases. | Thank you for the observation. We have clarified that the dataset includes both probable (clinical-epidemiological) and confirmed (laboratory-verified) cases, following national surveillance definitions. |
| - L193-194. Please mention and describe these univariate and multivariate techniques. | Thank you. We clarified that the univariate and multivariate techniques refer to Kulldorff’s scan statistics applied separately for each virus and jointly to detect co-circulation. |
| - L196. Please briefly describe the method and the assumptions it considers. | We have added a brief description of the scan statistic method and its assumptions to clarify the analytical approach used. |
| - L199. Which cylinder are the authors referring to? | Thank you. We clarified that the “cylinder” refers to the space-time scanning window used in Kulldorff’s method, where the base represents a geographic area and the height represents time. |
| - L211. Which specific window are the authors referring to? | The description of the formula was clarified |
| - L217. Please explain what I() means. - L217. Which program? What is the meaning of I() equal to 1? | We noticed that the equation was incomplete and have now included the missing information. |
| - L224. Which location are the authors referring to? | It was noted which comunas the mentioned population is located in. |
| - L229. Please describe how the maximum radius of the cylinder is considered. | The wording was revised for clarity. It is stated that the radius was one kilometer in all cases. |
| - L229-232. Please explain how and why those values were defined. | Estimations were performed using multiple parameters until the model with the greatest interpretability was identified. |
| **5. Discussion** |  |
| - L340. Please explain why in western comunes if figs. 2 and 3 show the clusters in the Northeastern comunas. | Thank you for pointing this out. We corrected the text to reflect that the clusters were primarily located in northeastern. The north orientation indicator was adjusted in the images. |
| - L341. How can the authors say that without a seasonal analysis? | We revised the sentence to clarify that no formal seasonality analysis was performed. The observation was based on visual inspection of the time series only. This study refers to cases, no seasonal analysis. |
| - L343-344. Please explain what a "similar trend" means. | Thank you. We revised the sentence to clarify what is meant by a "similar trend," specifically referring to the periodicity of dengue outbreaks observed both locally and nationally. |
| - L351. What do the authors mean by saying "this study showed a cyclical behavior until 2016"? That is not reported in the results. | Thank you for pointing this out. We have revised the sentence to clarify that our data reflects cyclical dengue peaks up to 2016, but not beyond, and that no clear cycles were observed after that year. |
| - L355. What do the authors mean by less rainfall? Less than what? Also, is Medellín a city with "less" rainfall? | Thank you for this important observation. We revised the sentence to clarify the reference to rainfall, specifying that the comparison was made relative to other regions in Colombia and to average conditions in endemic areas. |
| - L356-358. Please explain which Medellín's trends? The results do not provide any analysis of trend behavior to say that the increase in Chicungunya and Zika in the country reflects the trend in Medellín. | Thank you. We revised the sentence to avoid suggesting that national trends reflect local trends, since no formal trend analysis was conducted for Medellín. |
| - L359. Please explain what "discrepances" are being referring to. | Thank you. We have clarified the term “discrepancies” to specify that it refers to variations in reported of cases. |
| - L361-362. Please explain what the authors mean by this statement. | Thank you. We revised the sentence to clarify that the 2016 peak is highlighted due to the simultaneous co-circulation of all three arboviruses, which is relevant for understanding overlapping transmission dynamics. |
| - L372. Again, this type of statement cannot be concluded from this analysis. A trend analysis is needed to identify trends. | Thank you. We agree and have revised the sentence to avoid suggesting a trend without formal analysis, clarifying that the observation refers to a decrease in reported cases. |
| - L374. Wolbachia was not released in all comunas and not at the same time. Can this information help to understand the clusters? | Thank you. We agree and have clarified that Wolbachia was not released uniformly across all comunas. We now discuss how the phased implementation may help explain the spatial distribution of clusters. |
| - L383-389. Please describe the relevance of those other analyses performed at other sites to this analysis. Why are the values different? Any hypotheses? | Thank you. We clarified the relevance of these comparisons and proposed potential explanations for differences in cluster duration, including variations in surveillance systems, population density, vector control strategies, and timing of virus introduction. |
| - L391. This is the first time the "marginalized areas" are mentioned in this manuscript. The authors have not said anything about socioeconomical conditions so far to compare with national and local studies by others. | Thank you for your observation. We revised the paragraph to include specific information about the socioeconomic conditions of the clustered areas in Medellín, providing context for the comparison with national and local studies. |
| - L391-394. Why are those results different? Have conditions changed? Please contextualize those studies with yours and explain the source of the differences. | Thank you. We revised the paragraph to contextualize the differences between our findings and previous studies, and we suggest possible explanations such as urban expansion, changing transmission dynamics, and differential implementation of vector control measures. |
| - L396-397. Why is this study relevant here? Please contextualize with your study. | We revised the text to clarify the relevance of the cited study, emphasizing how its findings on recent case concentration in the northeastern zone of Medellín align with the clusters identified in our analysis. |
| - L400. It seems pretty obvious to me given that they are transmitted by the same mosquito. What may be interesting is to explaing why, according to the results of this analysis, there are clusters in which the three viruses do not co-circulate and cluster in which they do. | We revised the text to move beyond the common vector explanation and now discuss potential factors that may explain why co-circulation occurred in some clusters but not others. |
| - L403-404. This is not a conclusion of this work. | Thank you. We agree that this sentence extended beyond the scope of our findings. We revised it to focus only on the evidence supported by our analysis. |
| - L408. Please explain the specific scores. There are different quantifications and the range of values are different between them. | Thank you. We clarified that the scores refer to the Life Condition index, which ranges from 0 to 100, with lower values indicating worse living conditions. |
| - L409. Please explain what the reasons for the exception in Buenos Aires should be. | We have added a possible explanation for the exception observed in Buenos Aires, considering factors beyond socioeconomic status, such as connectivity and proximity to high-risk areas. |
| - L415. Please explain what the authors mean by "acceptance" in this context. | We revised the sentence to clarify that "acceptance" refers to community engagement and willingness to participate in vector control and prevention strategies. |
| - L416-417. The role of socioeconomic and environmental factors in dengue transmission is widely known. The relevant information is which specific factors drive that phenomena and how (the mechanistic processes). | We revised the sentence to specify which socioeconomic and environmental factors may influence dengue transmission in the study setting, and we briefly discuss possible mechanisms. |
| - L418-419. Please contextualize those results with your results. | We revised the sentence to relate the findings from Fortaleza to our own results, highlighting the similarities in the concentration of cases in low-income communities. |
| - L424-425. Please explain the assumption or references saying that climatic variations in Medellin are similar. This city has a complex topography that may result in significant climate differences across the territory. | We revised the sentence to remove the assumption of uniform climate and acknowledge Medellín’s topographic variability, which may influence local transmission conditions. |
| - L427-431. Please mention that the cited study considers data up to 2010. Conditions have likely changed in the last 15 years. Furthermore, explain the consequences and implications of those changes on your results. | We clarified that the cited study includes data only up to 2010, and we discussed how more recent environmental changes may influence current transmission patterns and our results. |
| - L431-433. Please explain why those results are relevant to your analysis. | We revised the text to explain how long-term climatic changes, although not directly analyzed in our study, may contribute to understanding the environmental context in which arboviral transmission occurs. |
| - L442. Most cases are suspected cases, and only a small number of records are associated with an ELISA or PCR test. | We revised the text to clarify some cases were clinically suspected, with a proportion confirmed by laboratory tests such as ELISA or PCR. |
| - L444-447. Please explain why identifying clusters remains crucial to understanding the spatial distribution of cases resolves the limitation that the recorded address corresponds to the patient residence rather the place of infection. | We clarified that, although cases are georeferenced by residence, identifying spatial clusters remains relevant for guiding local surveillance and public health interventions, since arboviral transmission is often associated with residential environments. |
| - L449. Please describe the limitations of the methods used. | We added a paragraph describing the methodological limitations of the scan statistic approach used in our analysis. |
| ***Minor comments** |  |
| 1. Abstract - L37. No need to mention the programming language. - L41. Please mention that those cases correspond to potential cases. | We removed the mention of the programming language from the abstract and clarified that the cases correspond to probable and confirmed reports, in line with national surveillance definitions. |
| 2. Introduction - L109-110. Prevalence of mosquito vectors is not an environmental factor. Environmental factors drive mosquitoes prevalence.  - L152-180. I do not consider those paragraphs relevant here. I suggest moving that information to an appendix or just writing the appropriate references. | - L109-110: We revised the sentence to clarify that environmental factors influence the presence and abundance of mosquito vectors, rather than being synonymous with vector prevalence.  -L152-180: We have summarized the case definitions to improve readability while retaining essential information. Full diagnostic criteria are now referenced and may be consulted in the corresponding official guidelines. |
| **3. Methods.** |  |
| - L120. Please explain why this is an 'ecological study'. | We have clarified that the study is ecological because it analyzes aggregated data at the neighborhood level rather than individual-level data. |
| - L133. What do the authors mean by LO and LN? | We have clarified that “LO” and “LN” refer to west longitude and north latitude, respectively. |
| - L133-135. Please provide the appropriate reference. | We reviewed an appropriate reference to support the geographic description of Medellín. |
| - L140-146. I do not consider this is to be appropriate place for the ethics statement. | We agree and have relocated the ethics statement to a separate section at the end of the methods, in accordance with standard publication practices. |
| - L150. It is probably not "in Colombia" but for the INS. Please check. | We revised the sentence to clarify that the case definitions are established by the Instituto Nacional de Salud (INS), in Colombia |
| - L150-152. Please provide the appropriate reference. | We have added the official guidelines from the Instituto Nacional de Salud (INS) as a reference for the case definitions used. |
| - L162. I think it means >=38.5 deg C. | The procedural part was summarized, so the signs and symptoms were not included in the description. |
| - L188. Consider avoiding this type of sentence and going straight to the point. | We have rephrased the sentence to make it more concise and direct, as suggested. |
| - L201. Please refer to it as an equation instead of a formula and cross-reference it. | We revised the text to refer to it as an equation and included an in-text cross-reference. |
| - L203. Please explain here how E is determined. | E represents the number of expected cases within the cluster. Kulldorff’s descriptive analysis explains how these expected values are calculated based on the distribution of the population at risk." |
| - Eq. 2 and 3 are not necessary. They are widely known. | They are retained to ensure clarity in the procedures used to obtain the relevant indicators. |
| - L236-237. In my opinion, this not relevant. | They are retained to ensure clarity in the procedures. |
| **4. Results** |  |
| - L250. Why are you referring to incidence if the figure shows cases. | We revised the wording to refer to case counts instead of incidence, as the figure presents absolute numbers rather than population-based rates. |
| - L276. How can people outside the country recognize the "Comunas". Please cross-reference the figures before starting to describe them. Also, in the figures is not possible to identify the text due to the poor quality. | We clarified the meaning of "comunas" for international readers, added figure cross-references before the descriptive section, and improved the figures resolution to ensure that all labels and text are clearly readable. |
| **5. Discussion** |  |
| - L348-349. Please provide the appropriate reference | We added an appropriate reference supporting the cyclical pattern of dengue incidence observed every two to three years in Colombia. |
| - L363. This statement is quite obvious. | We revised the sentence to provide a more specific interpretation related to vector dynamics in the study context. |
| - L365. Explain that Antioquia is an administrative division with Medellín inside. | We have clarified that Antioquia is the department where Medellín is located. |
| - L407-408. Please mention those "previous reports". | We revised the sentence to specify the sources of the “previous reports” mentioned, including references that document the socioeconomic conditions of the affected areas. |
| - L409. Please define what the authors mean by "district" here. | We clarified that “district” refers to *comunas*, which are official administrative divisions within the city of Medellín. |
| - L412. Please explain why the areas are important here. | We revised the sentence to clarify that the size and population density of these areas are relevant because they may influence arbovirus transmission dynamics and inform intervention planning. |
| *Figures* - Figure 1. Panels A and B show repetitive information. Standarized cases are sufficient to recognize the times with the maximum number of cases. - Figure 1B. y-label corresponds to the standarized number of cases, not number of cases. - Figure 1. Please remove the days in the y-marks. - Figure 2 and 3. Please avoid the "Notes". Describe what each panel means in the main legend text. - Figure 2 and 3. Please correct the direction of the North arrow. - Figure 2 and 3. Please add what the divisions mean. - Figure 2 and 3. Please remove 'na' in the legend. | The standardized figure has been retained.  Labels and descriptions were improved, and the requested details have been organized accordingly. |
| *Supplementary material* - Consider reducing the number of supplementary figures to those that are really necessary. - Please describe what is in each supplementary figure with the different panels. | Six supplements were removed; this version includes six revised supplementary figures. The panels are now better described, and a more detailed explanation has been incorporated into the main text. |

**Reviewer 3**

| **Comment** | **Response to Reviewer** |
| --- | --- |
| **Methods** |  |
| The github repository that is supposed to contain the data used in this analysis is missing. | The github repository is: https://github.com/jemilios/Spatiotemporal |
| **Results** |  |
| Results match the analysis plan. The presentation of some of the results could be clearer. Details for how to improve them is provided in the general comments. | We have revised the presentation of the results to improve clarity, following the specific suggestions provided in the general comments. These adjustments aim to enhance the interpretability and coherence of the findings in alignment with the analysis plan. |
| **Summary and General Comments** |  |
| This manuscript explores the spatiotemporal distribution of three arboviruses in Medellin, Colombia over the past decade. They provide evidence of co-circulation of all three pathogens and overlapping hotspots of dengue and Zika incidence shortly after the introduction of Zika in 2015-2016. The analysis is fairly limited and does not attempt to explain the timing of these clusters or the potential drivers of the observed spatial patterns. There are several issues that should be addressed to strengthen the results of this study.  Despite being described as a spatiotemporal analysis, there is no attempt to explain the observed patterns beyond the identification of several clusters of transmission and co-transmission. This analysis would be considerably strengthened if you were able to examine the association between these clusters and relevant socioeconomic and environmental factors at the neighborhood or district(communa) level.  Given the issues of underreporting and the potential for misdiagnosis among the three arboviruses with probable cases, it would be helpful to know what % of cases for each pathogen were confirmed versus probable. And if there is enough data, it could be useful to run the analysis on only confirmed cases to see if the patterns hold up.  There should also be some additional discussion of the potential for misdiagnosis and its implications on the observed spatiotemporal patterns. Have there been any seroprevalence studies for any of these arboviruses that would give an indication of the infection rate and how it compares to observed incidence? I also wonder about the large increase in dengue cases that overlaps with the Zika outbreak. Could this be do to changes in surveillance during this time period. In most localities there were was increased surveillance and reporting of disease due to the risk of microcephaly and other Zika complications.  The analysis is of a single city in Colombia, but most of the Introduction is a very broad overview of the three pathogens and their recent epidemiology in Latin America. I think the Intro would benefit from at least a paragraph on what is known about the intra-city spatiotemporal patterns of these arboviruses and what the potential drivers of these patterns are in the region. As well as why this scale of analysis, as opposed to a broader regional or country-level analysis, is important.  It would be helpful to have a map of the mean annual incidence rates at the neighborhood level for each pathogen. Since the existing maps don’t show RR outside the clusters it is hard to visualize the overall pattern of incidence in the city. | Thank you for your insightful review. We have incorporated your suggestions to improve the manuscript:  The Introduction now includes a focused overview of intra-urban arboviral transmission and its drivers in Colombia and Latin America.  The Discussion explores potential explanations for the observed clusters, including socioeconomic vulnerability, entomological risk, and public health interventions.  We added a paragraph on the limitations of using probable vs. confirmed cases, with a breakdown by pathogen and a call for future seroprevalence studies.  We acknowledged the role of enhanced surveillance during the Zika epidemic in the observed dengue increase. |
| **Minor comments:** |  |
| Line 65 of Author summary: “This analysis addresses a critical gap in understanding the intra-urban spread of arboviruses in tropical cities”. I don’t see how this analysis addresses intra-urban spread as there is no analysis or discussion of pathogen spread from one location to another. | We agree and have revised the sentence to better reflect the nature of our analysis, focusing on intra-urban spatial distribution rather than pathogen spread. |
| Lines 96-97: “For instance, …” – The use of this phrase is misplaced as this sentence is unrelated to the previous sentence. | We revised the sentence to remove the misplaced connector and improve logical flow. |
| Lines 221-222: Why do c and p represent the number of cases and population size in each district (communa) and not at the neighborhood-level as I thought that was the level of analysis being conducted? | We clarified that both the number of cases (c) and population size (p) were calculated at the neighborhood level, in line with the unit of analysis used throughout the study. |
| Line 229: Can you explain further how considered the maximum radius of the cylinder? I didn’t see any mention in the Results to an examination of different values for the cylinder radius. | We acknowledge that the original wording was ambiguous. We have clarified in the revised manuscript that all analyses were conducted using a one-kilometer radius for the scanning cylinder. |
| Line 272 – Dengue spatial and temporal distribution: I think it needs to be made a bit clearer that the main result of 5 dengue clusters is when the scan is done for entire time period. Initially I was confused about the difference between this result and the mention of differing number of clusters for the shorter time periods mentioned at the end of this section. It might also be useful to state either here or in the Methods why these specific additional time periods were chosen. | We clarified that the five clusters correspond to the full-period analysis. |
| Line 285: Why does this sentence start with “Conversely”? It does not seem to contradict the findings in the previous section. | We agree that “Conversely” was not appropriate in this context and have replaced it with a more accurate transition. |
| Lines 323-324: You give the length of clusters 3 and 5, but not the other three. | We have added the duration of all five dengue clusters to ensure consistency in reporting. |
| Figure S12 – The RR displayed in this figure doesn’t seem to match the RR values in Fig 2D. Is that due to the differing time periods in the two maps? | This figure was eliminated |
| Line 337-338: It is not clear what this sentence means or how the identification of dengue clusters is evidence of cyclical viral activity. | We revised the sentence to clarify that the observed periodic peaks suggest cyclical activity, rather than being a direct result of the cluster analysis. |
| Line 351: It would be useful to also mention the behavior observed post-2016. | We have included a sentence summarizing the dengue pattern observed after 2016 to provide a more complete temporal context. |
| Line 357: CHIKV wasn’t present before 2014, so I wouldn’t call the observed pattern “increased incidence”, especially since incidence declined strongly by the end of 2017. | We have rephrased the sentence to more accurately describe the temporal pattern of chikungunya in Medellín, avoiding the misleading term “increased incidence” for a newly introduced virus. |
| Line 359: Discrepancies in what? | We clarified the sentence to specify the nature of the discrepancies being referenced. |
| Lines 371-372: Please edit this sentence for grammar and clarity. | We have revised the sentence to improve grammar and clarity. |
| Line 378: I assume you are referring to the COVID-19 pandemic? | We clarified the reference to specify the situation. |
| Lines 392-394: Can you provide some explanation for why prior studies found differing patterns to the current study? | We have added an explanation discussing potential reasons for differences in findings between our study and previous research, including geographic scale, study period, intervention timing, and contextual factors such as immunity and socioeconomic conditions. |
| Lines 392-394: Can you provide some explanation for why prior studies found differing patterns to the current study? | We have revised the text to include potential explanations for the differences between our findings and those of previous studies, including variations in spatial resolution, timing of virus introduction, public health interventions, and local contextual factors. |
| Lines 424-425: What findings remain significant? You didn’t look at climate variability in your analysis. | We agree with your point and have revised the sentence to clarify that the discussion on climate is speculative and not based on our own empirical findings. |
