# Supplementary material for: Spatio-temporal analysis of the distribution and co-circulation of dengue, chikungunya, and Zika in Medellín, Colombia, from 2013 to 2021
Source: PLoS Negl Trop Dis. 2025 Sep 5;19(9):e0013470. doi: 10.1371/journal.pntd.0013470 (PMC12425392; doi:10.1371/journal.pntd.0013470)
Supplement: Attachment — Submitted filename: Response_to_Reviewers_auresp_2.docx [file pntd.0013470.s008.docx]

July 22, 2025

**Shaden Kamhawi**

Editor-in-Chief

PLOS Neglected Tropical Diseases

Dear Editor and reviewers,

We sincerely appreciate the time and effort that you and the reviewers have dedicated to evaluating our manuscript, *“Spatio-Temporal Analysis of the Distribution and Co-Circulation of Dengue, Chikungunya, and Zika in Medellín, Colombia, from 2013 to 2021.”* We are grateful for the constructive feedback, which has helped us refine and strengthen the manuscript.

Below, we provide a detailed point-by-point response to each of the reviewers’ comments. For clarity, all revisions in the manuscript are highlighted in turquoise blue.

We hope that our responses and the revised version of the manuscript meet the reviewers’ expectations and that the article is now suitable for publication in *PLOS Neglected Tropical Diseases*. Please do not hesitate to contact us should any further modifications be required.

**Reviewer 1**

| **Comment** | **Response to Reviewer** |
| --- | --- |
| **Methods** |  |
| Related to hypothesis: It is not clear why authors hypothesized that socioeconomically disadvantaged conditions presented specifically at the northeastern districts render the areas more susceptible to spatio-temporal cluster. Is it a results-driven hypothesis? | Thank you for this valuable observation. We agree that the original hypothesis could be misinterpreted as results-driven. To address this, we have removed the explicit hypothesis from the introduction and methods. Instead, we now refer more subtly to the observed concentration of clusters in socioeconomically disadvantaged northeastern districts in the discussion, framing it as a hypothesis that should be further explored by future studies. We have clarified that this was not derived from the analysis itself. |
| Though the manuscript improved, I believe that the fundamental concerns of the methodology were not appropriately resolved: The work does not make analysis aimed to understand the nature of the clusters. So, instead of providing information leading to potential improvement of control activities, it presents an analytical narrative of historical reports. Authors claimed that the purpose of these analyses is to “enhance future outbreak predictions, enabling targeted resource allocation for prevention and control efforts”. All clusters that authors found were tied to a specific epidemic. I wonder how reproducible the found clusters are if only one epidemic was analyzed. If similar clusters would be found through multiple epidemics, it could be stated that reproducible pattern in time is happening and enhanced predictions could arise. For example, the found clusters for Zika and Chikungunya are temporally located within epidemics that happened due to introductions into a naïve population. There haven’t been more epidemics since then as the populations is no longer naïve. How these results improve control of these diseases? | We appreciate this important observation. Our analysis was descriptive by design and does not aim to model the drivers of clustering or predict future outbreaks. While Zika and chikungunya clusters reflect single, introduction-driven epidemics, our findings for dengue—an endemic virus—do reveal recurrent clusters in the same northeastern districts, suggesting persistent transmission hotspots. These results can still inform targeted control strategies, even if predictive capacity is limited. We have revised the discussion to clarify these points and avoid overstating the implications. |
| **Results** |  |
| Results matched the analysis plan. Besides the big improvement of the manuscript, I think the problem is the plan itself. As it's mentioned in the previous review: "While these type of studies are locally relevant, I believe they may have limited appeal to a broader audience as it is limited only to the descriptive level". There's room for a lot of improvements to the work. | We appreciate this thoughtful reflection. We acknowledge that the study is limited to a descriptive spatio-temporal analysis and agree that this constrains its broader generalizability. However, we believe it provides important foundational evidence at the intra-urban level in a hyperendemic city, serving as a baseline for future studies that incorporate explanatory and predictive modeling. We have adjusted the framing of the manuscript to reflect this scope more accurately and avoid overstating its impact. |
| **Conclusions** |  |
| Conclusions are supported by data and results. Some limitations are clearly described. My biggest concerns are related to public health relevance: the study is highly local and results are not extrapolable to other settings or times. | Thank you for your comment. We agree that the study is geographically specific and its findings are not directly generalizable to other settings or future outbreaks. However, we believe that describing fine-scale spatio-temporal dynamics in hyperendemic urban contexts like Medellín provides valuable insights for local health authorities and serves as a model for similar cities facing the challenge of arboviral co-circulation. We have revised the discussion and conclusions to better reflect this scope and to emphasize its utility for local planning rather than broad extrapolation. |
| **Editorial and Data Presentation Modifications?** |  |
| Line 103: Improve references and data of asymptomatic infections. | We revised line 103 to include updated and specific estimates of asymptomatic infections based on recent meta-analyses: ~59% for dengue, 25–40% for chikungunya, and 33–88% for Zika. References from 2023–2024 have been added to support these figures. |
| Line 221: A reference should be used when mentioned SaTScan for first time. Also, the version of the software used should be reported. | We have added a reference for SaTScan upon first mention and included the version used (v10.1). This has been corrected in the Methods section. |
| The sentence “The city's administration divides Medellín's territory into 21 units known as 'Comunas'.” Is repeated | We have revised the sentence for clarity |
| Improve position/size of legends as they overlap maps. | Thank you for your suggestion. We attempted to reduce the size and reposition the legends to avoid overlap; however, due to technical limitations and the need to retain key geographic elements—such as *comuna* names and neighborhood codes—it was not feasible to make significant changes without compromising the readability and spatial resolution of the maps. These elements are essential to convey the high level of geographic detail and the intra-urban heterogeneity of arbovirus distribution. We prioritized visual clarity while preserving these important references. |
| **Summary and General Comments** |  |
| The manuscript is improved in regards to basic elements exposed by reviewers. But in my point of view, fundamental improvements should be done, i.e. take the data to broader analysis and results, so the analysis can acquire bigger relevance and can potentially be generalizable to other settings. | Thank you for your thoughtful assessment. We acknowledge that broader analytical approaches—such as explanatory or predictive modeling—would enhance the generalizability and external relevance of the study. However, our objective was to establish a detailed spatio-temporal description of arbovirus clustering in Medellín, which, to our knowledge, had not been previously documented at this scale. We believe these findings provide an essential foundation for future studies aiming to explore causal mechanisms or conduct comparative analyses across urban settings. We have clarified this in the discussion to better reflect the study’s scope and potential. |
